# Neuroimaging Studies of Brain Structure in Older Adults with Bipolar Disorder: A Review

**DOI:** 10.20900/jpbs.20220006

**Published:** 2022-08-25

**Authors:** Niroop Rajashekar, Hilary P. Blumberg, Luca M. Villa

**Affiliations:** 1 Department of Psychiatry, Yale University School of Medicine, New Haven, CT 06511, USA; 2 Department of Radiology and Biomedical Imaging, Yale University School of Medicine, New Haven, CT 06520, USA; 3 Child Study Center, Yale School of Medicine, New Haven, CT 06519, USA; 4 Department of Psychiatry, University of Oxford, Oxford, OX37JX, UK

**Keywords:** bipolar disorder, aging, gray matter, white matter, neuroimaging, brain structure

## Abstract

Bipolar disorder (BD) is a common mood disorder that can have severe consequences during later life, including suffering and impairment due to mood and cognitive symptoms, elevated risk for dementia and an especially high risk for suicide. Greater understanding of the brain circuitry differences involved in older adults with BD (OABD) in later life and their relationship to aging processes is required to improve outcomes of OABD. The current literature on gray and white matter findings, from high resolution structural and diffusion-weighted magnetic resonance imaging (MRI) studies, has shown that BD in younger age groups is associated with gray matter reductions within cortical and subcortical brain regions that subserve emotion processing and regulation, as well as reduced structural integrity of white matter tracts connecting these brain regions. While fewer neuroimaging studies have focused on OABD, it does appear that many of the structural brain differences found in younger samples are present in OABD. There is also initial suggestion that there are additional brain differences, for at least a subset of OABD, that may result from more pronounced gray and white matter declines with age that may contribute to adverse outcomes. Preclinical and clinical data supporting neuro-plastic and -protective effects of mood-stabilizing medications, suggest that treatments may reverse and/or prevent the progression of brain changes thereby reducing symptoms. Future neuroimaging research implementing longitudinal designs, and large-scale, multi-site initiatives with detailed clinical and treatment data, holds promise for reducing suffering, cognitive dysfunction and suicide in OABD.

## INTRODUCTION

Bipolar disorder (BD) is a common disorder, with an estimated lifetime prevalence of 2.4% [1]. In addition to the profound suffering from its symptoms, BD carries high rates of comorbidity and suicide [2]. While the onset of BD typically occurs before the age of 25 years [3], it persists throughout the life-course and, for at least a portion of those with the disorder, the illness evolves with age, being associated with a greater risk for cognitive impairment, dementia, and an especially high rate of suicide ideation and attempts in older adults with BD (OABD) [4–7]. Compared to BD in younger adults, BD in older adults has been associated with more severe depressive episodes [8], and greater rates of episode recurrence [9,10]. These severe clinical outcomes in OABD, alongside rapidly aging populations across the globe [11], give new impetus to understand aging and the brain in BD [12]. Here we define OABD as individuals with BD and an age of 40 years or more, as an age of 40 years provides a more comprehensive picture of BD during later life that is more able to encompass individuals with a later onset of the illness [10]. Moreover, as this is a review of structural neuroimaging, it considers work suggesting that the brain begins to undergo age-related gray and white matter declines during the fourth decade of life [13,14], which is especially pertinent to OABD given hypotheses suggesting that it may be characterized by accelerated aging processes [15–17].

Numerous neuroimaging studies of BD have characterized differences in brain structure, although participants have primarily been in the second through fifth decades of life [18]. There is a growing body of research examining brain structure in OABD and potential effects of aging processes. This mini-review will explore the neuroimaging literature on gray matter (GM) and white matter (WM) findings in OABD, their relationship with medication use and aging processes, and future directions for the field.

## GRAY MATTER IN BIPOLAR DISORDER

Three key metrics have been used to assess GM structure in BD: volume and its components in the cortex, thickness and surface area. Cortical thickness is defined as the distance between the cortex’s inner GM-WM boundary and outer pial GM surface; cortical surface area is defined as the surface area of the cortex’s outer pial surface. As cortical thickness and surface area have differing genetic and environmental contributions [19], investigations of these GM metrics in BD are providing intriguing insights into the underlying neurobiology of the illness. Measures of cortical gyrification have also been studied in BD, but to a lesser extent and rarely in OABD.

The largest structural neuroimaging literature in BD is on studies of GM volume in individuals mostly below the age of 40 years, with findings converging in subcortical and cortical areas that subserve emotion processing and regulation. Lower subcortical GM may be a particularly early difference associated with BD. In adolescents and young adults with BD, lower GM volume in the amygdala is one of the most consistently reported results [20–23]. GM differences have also been reported in other subcortical regions, albeit with some variable findings, such as the hippocampus, striatum, thalamus and cerebellum [23–29]. Lower cortical GM in ventral prefrontal areas are also some of the most frequently reported findings in BD [30], and progressive decreases in adolescents and young adults with BD suggest an underlying neurodevelopmental pathophysiology [31,32]. GM volume decreases have also been reported in lateral and medial dorsal frontal cortices, and, although less frequently studied, in insular and temporopolar cortices [33–38]. Lower cortical thickness has been reported in these regions, while cortical surface area differences have been inconsistent [34,36,37,39,40]. These findings suggest that in BD, anterior cortical GM reductions are predominantly driven by factors that lower cortical thickness, providing a potential lead about underlying pathophysiology.

While there are substantially fewer neuroimaging studies focusing on OABD, most investigations of OABD echo the results in younger samples as they show lower GM volume in structures that subserve emotion processing and regulation, including the dorsolateral and ventromedial prefrontal cortex, hippocampus, amygdala, and striatal regions [41–45]. However, there are studies with conflicting and negative findings [46–49], and it is noteworthy that the majority of the samples in these neuroimaging studies have been comprised of 20 or fewer individuals with BD [42–44,47–49]. In addition to differences across studies in imaging methods, subject heterogeneity may have contributed to the discrepant findings.

Given that OABD is associated with a greater risk for cognitive impairment, a small number of studies have attempted to investigate how GM properties relate to cognition in OABD, however no consistent relationships have so far been detected [43,46,49]. Similarly, given the greater risk for dementia associated with OABD, two studies have directly compared the GM properties of individuals with frontotemporal dementia and OABD. The disorders showed similar GM reductions within insular and anterior cingulate cortices, but also showed differences in the involvement of regions within prefrontal and posterior cortices, with OABD showing more prominent GM reductions within the ventrolateral prefrontal cortex, and individuals with frontotemporal dementia showing more prominent GM reductions within dorsolateral prefrontal and orbitofrontal cortex, as well as temporal, occipital and parietal cortices [42,49]. However, these studies were limited in sample size, including only 13-16 individuals with BD, so should be viewed with caution. Future studies directly investigating commonalities between OABD-related and dementia-related GM reductions are warranted, as they may elucidate why OABD confers a greater risk for dementia.

## WHITE MATTER IN BIPOLAR DISORDER

WM deficits are increasingly implicated as integral in the pathophysiology of BD, with supportive lines of evidence from neuroimaging studies [50], such as initial studies consistently reporting greater numbers of WM hyperintensities within periventricular and deep WM [51–53] in both younger and older individuals with BD [54].

More recently, diffusion tensor imaging (DTI) has been used to study BD, due to its sensitivity to the microstructural integrity of WM tracts. A commonly used metric from DTI is fractional anisotropy (FA). FA measures whether water diffusion within WM tracts is more restricted towards moving in the direction of the tracts with high FA inferring greater water diffusion along the WM tract and therefore greater WM integrity. DTI-based studies have shown lower FA across numerous WM tracts in BD [50,55–57], particularly within the frontotemporal emotion regulation system [30]. The corpus callosum is one of the WM tracts most studied, with findings in BD reported most often in the genu, which provides connections between the right and left ventral prefrontal cortices [58–64]. Of tracts providing intra-hemispheric connections, in BD, lower FA has been found in the uncinate fasciculus [65–69], a tract that provides the major connections between the ventral prefrontal cortex and the amygdala [70]. Progressive differences in FA in the uncinate fasciculus, observed in a longitudinal study of adolescents and young adults with BD, suggest developmental contributions to uncinate WM pathology [50].

While a greater number of WM hyperintensities has frequently been reported in OABD and in younger samples, it appears that WM hyperintensities may be more abundant in OABD particularly within deep WM [54]. Furthermore, the causes underlying WM hyperintensities in BD may be related to age and comorbidities. In OABD, and particularly in those with a later onset of BD, it is thought that cardiovascular and metabolic comorbidities that have high rates in OABD [10,71,72], may contribute to WM hyperintensities [54]. However, more studies directly assessing these associations, and of the specific mechanisms involved, in OABD are needed [73].

Furthermore, studying OABD using DTI methods is a burgeoning area, with several studies showing FA decreases in similar areas as in younger samples with BD, including ventral corpus callosum, and uncinate fasciculus [74,75]. In addition to contributions to emotion dysregulation in BD, contributions to cognitive deficits are implicated by negative correlations between cognitive performance across multiple domains, including working memory, information processing, executive function, and attention, with FA in the corpus callosum and uncinate fasciculus [76–78]. As cognitive decline is a feature for some individuals with BD during later life, that causes suffering, impaired functioning, and is associated with poor prognosis [79], elucidating disturbances in these key WM tracts may be integral to preventing some of the severe clinical outcomes associated with BD during later life.

## MEDICATIONS AND BRAIN STRUCTURE IN BIPOLAR DISODER

An intriguing avenue of research is on the potential for mood-stabilizing medications to have neuroplastic and neuroprotective effects. This is supported by preclinical data demonstrating that mood-stabilizing medications, with lithium being most studied, upregulate molecular factors that promote plasticity and down-regulate factors that promote cell death [80]. Neuroimaging findings in individuals with BD are consistent with these effects, such as lithium-associated increases in GM volume of the amygdala and hippocampus [45,81,82], and in WM FA [57,83–86]. Moreover, in OABD, there is a growing body of work suggesting that lithium use is associated with a lower risk for developing dementia [5,87]. Together, these findings suggest that lithium may have neuroplastic and neuroprotective effects within the brain in OABD that could potentially reverse and/or prevent progression of detrimental age-related processes [45,81,82]. However, randomized control trials are required to confirm these effects.

While lithium has been the most studied medication in imaging studies of BD, varying effects of other medications have been reported. For example, antipsychotic medications, which are highly prescribed for OABD [88], have been associated with more extensive GM reductions in younger individuals with BD [23,26,34,89]. The relationship between antipsychotic medication use and WM structure in BD, however, is less clear with some studies finding reduced FA within the corpus callosum [57] and others finding no effects [69,83,90]. Far less work has focused on brain structure and antipsychotic medication use in OABD. One study of a sample of 54 individuals with BD found that antipsychotic medication use was associated with lower overall GM volume across the brain and particularly within the hippocampus [91], however, it is yet to be replicated. Moreover, randomized controlled trials that longitudinally investigate the effects of antipsychotic medications on brain structure in BD have not been conducted, and it is therefore difficult to establish causality.

## AGING PROCESSES AND BRAIN STRUCTURE IN BIPOLAR DISORDER

While differences at the group-level in GM and WM structure appear to be present in OABD, it is still unclear whether the illness is associated with differences in brain aging processes. It has been proposed that BD is characterized by accelerated biological aging [15–17], a hypothesis supported by findings in BD of more pronounced markers of biological aging such as shorter telomeres [92], greater markers of oxidative stress [93], and reduced mitochondrial DNA copy numbers [94]. Moreover, findings of group differences showing lower GM properties and FA in BD have been used to suggest that accelerated aging of the brain may also be a characteristic of the illness [15–17], given that these structural properties decline during typical aging [95–99]. However, as many of these differences in GM and WM have also been found in younger samples, their causes may be neurodevelopmental rather than related to aging.

While few in number, studies investigating the relationship between brain structure and age in BD have been equivocal, with most failing to find age-related differences associated with the illness [47,100–102]. The largest longitudinal investigation of GM structure in BD, involving a sample of 307 individuals with BD, who had a mean age of 40 years, recently found no evidence of accelerated cortical thinning in BD. Moreover, studies investigating age-related differences in DTI-based WM properties in adults with BD have been unclear, with some failing to identify age-related differences associated with the illness [101,103]. More recent brain-based age-prediction models suggest that BD may show subtle indications of greater brain age [104,105] though these are yet to be supported by longitudinal evidence, which are required to support hypotheses surrounding effects of aging processes in BD.

The age of BD onset may be a particularly salient factor in attempting to understand brain structure and aging, specifically in OABD. The definition of late-onset BD has varied substantially across studies, with studies defining it as occurring after the age of 60 years [53], 50 years [106,107], 40 years [10], and others as low as in the late twenties [108]. Given that in around 50% of cases, the onset of BD occurs below the age of 25 years [3], an age of onset above 25 years does appear to mark a meaningful difference in onset that may have a different underlying neurobiology [3,109,110], and although prior studies have tended to use later age cutoffs, findings from studies using younger age of onset cutoffs are emerging [108,110–112]. A later onset of BD has frequently been associated with greater cognitive impairment across many cognitive domains in OABD [4,113–117], and it has been suggested that a later onset of BD may be associated with a greater level of WM differences, with one study finding that late onset BD was associated with a greater number of WM hyperintensities and another finding that a later onset of BD was associated with lower FA within multiple WM tracts, including the corpus callosum [53,54,107,108,118]. To date, few studies have investigated the effects of age of BD onset on GM properties in OABD. Interestingly, findings have been identified using measures of gyrification, with two studies showing that a later onset of BD was associated with lower levels of cortical folding [111,112]. This is intriguing given that lower levels of cortical folding have previously been proposed as a potential marker for Alzheimer’s disease [119,120], and suggests that future imaging work should consider other structural neuroimaging metrics such as gyrification measures when studying OABD. Together, while few in number and limited by small samples, these studies implicate age of BD onset as a key clinical factor that may interact with aging processes within the brain in OABD and may be involved in the severe clinical outcomes experienced by some individuals with BD during later life, such as cognitive impairment.

## FUTURE DIRECTIONS

Crucial areas for further study of OABD include neuroimaging investigations of larger samples of individuals with comprehensive demographic and clinical data, and with longitudinal designs necessary to clarify aging-related processes. Moreover, given the heterogeneity of BD, in both its clinical presentations and in its outcomes, more work is needed to identify differences between individuals with BD, to find neurobiological mechanisms that are specific to key clinical phenotypes of the illness during later life, such as differences associated with BD-type (e.g., BDI or II), presence of psychosis, cognitive impairment, age of illness onset, and risk for dementia. Promising new initiatives that involve large scale, international multi-site data harmonization approaches, such as in the Global Aging and Geriatric Experiments in BD (GAGE-BD) consortium [121], are likely to be indispensable in harnessing datasets with enough clinical detail and statistical power to further our understanding of differences between individuals with BD during later life, and to provide more specific and effective interventions.

## ABBREVIATIONS

BD: Bipolar Disorder
OABD: Older Adults with Bipolar Disorder
GM: Gray Matter
WM: White Matter
DTI: Diffusion Tensor Imaging
FA: Fractional Anisotropy

## References

[1] Merikangas KR, Jin R, He JP, Kessler RC, Lee S, Sampson NA, Prevalence and correlates of bipolar spectrum disorder in the world mental health survey initiative. Arch Gen Psychiatry. 2011 Mar;68(3):241–51. doi: 10.1001/ARCHGENPSYCHIATRY.2011.12 21383262 PMC3486639

[2] Dong M, Lu L, Zhang L, Zhang Q, Ungvari GS, Ng CH, Prevalence of suicide attempts in bipolar disorder: a systematic review and meta-analysis of observational studies. Epidemiol Psychiatr Sci. 2019 Oct 25;29:e63. doi: 10.1017/S2045796019000593 31648654 PMC8061290

[3] Baldessarini RJ, Tondo L, Vázquez GH, Undurraga J, Bolzani L, Yildiz A, Age at onset versus family history and clinical outcomes in 1,665 international bipolar-I disorder patients. World Psychiatry. 2012;11(1):40–6. doi: 10.1016/j.wpsyc.2012.01.006 22295008 PMC3266753

[4] Samamé C, Martino DJ, Strejilevich SA. A quantitative review of neurocognition in euthymic late-life bipolar disorder. Bipolar Disord. 2013 Sep;15(6):633–44. doi: 10.1111/bdi.12077 23651122

[5] Velosa J, Delgado A, Finger E, Berk M, Kapczinski F, de Azevedo Cardoso T. Risk of dementia in bipolar disorder and the interplay of lithium: a systematic review and meta-analyses. Acta Psychiatrica Scandinavica. 2020 Jun;141(6):510–21. doi: 10.1111/acps.13153 31954065

[6] O’Rourke N, Heisel MJ, Canham SL, Sixsmith A. Predictors of suicide ideation among older adults with bipolar disorder. PLoS One. 2017 Nov 16;12(11):e0187632. doi: 10.1371/journal.pone.0187632 29145409 PMC5690620

[7] Aizenberg D, Olmer A, Barak Y. Suicide attempts amongst elderly bipolar patients. J Affect Disord. 2006 Mar 1;91(1):91–4. doi: 10.1016/j.jad.2005.12.013 16434107

[8] Kessing LV. Diagnostic subtypes of bipolar disorder in older versus younger adults. Bipolar Disord. 2006 Feb;8(1):56–64. doi: 10.1111/j.1399-5618.2006.00278.x 16411981

[9] Angst J, Preisig M. Course of a clinical cohort of unipolar, bipolar and schizoaffective patients. Results of a prospective study from 1959 to 1985. Schweizer Arch fur Neurol und Psychiatr. 1995;146(1):5–16. 7792568

[10] Sajatovic M, Strejilevich SA, Gildengers AG, Dols A, Al Jurdi RK, Forester BP, A report on older-age bipolar disorder from the International Society for Bipolar Disorders Task Force. Bipolar Disorders. 2015 Nov;17(7):689–704. doi: 10.1111/bdi.12331 26384588 PMC4623878

[11] Kanasi E, Ayilavarapu S, Jones J. The aging population: demographics and the biology of aging. Periodontol 2000. 2016 Oct 1;72(1):13–8. doi: 10.1111/PRD.12126 27501488

[12] Coryell W, Fiedorowicz J, Solomon D, Endicott J. Age Transitions in the Course of Bipolar I Disorder. Psychol Med. 2009 Aug;39(8):1247. doi: 10.1017/S0033291709005534 19335937 PMC3551474

[13] Bartzokis G, Lu PH, Heydari P, Couvrette A, Lee GJ, Kalashyan G, Multimodal magnetic resonance imaging assessment of white matter aging trajectories over the lifespan of healthy individuals. Biol Psychiatry. 2012 Dec 15;72(12):1026–34. doi: 10.1016/j.biopsych.2012.07.010 23017471

[14] Bartzokis G, Beckson M, Lu PH, Nuechterlein KH, Edwards N, Mintz J. Age-related changes in frontal and temporal lobe volumes in men: a magnetic resonance imaging study. Arch Gen Psychiatry. 2001;58(5):461–5. doi: 10.1001/ARCHPSYC.58.5.461 11343525

[15] Fries GR, Zamzow MJ, Andrews T, Pink O, Scaini G, Quevedo J. Accelerated aging in bipolar disorder: A comprehensive review of molecular findings and their clinical implications. Neurosci Biobehav Rev. 2020 May;112:107–16. doi: 10.1016/j.neubiorev.2020.01.035 32018037

[16] Rizzo LB, Costa LG, Mansur RB, Swardfager W, Belangero SI, Grassi-Oliveira R, The theory of bipolar disorder as an illness of accelerated aging: Implications for clinical care and research. Neurosci Biobehav Rev. 2014 May;42:157–69. doi: 10.1016/j.neubiorev.2014.02.004 24548785

[17] Simon NM, Smoller JW, McNamara KL, Maser RS, Zalta AK, Pollack MH, Telomere Shortening and Mood Disorders: Preliminary Support for a Chronic Stress Model of Accelerated Aging. Biol Psychiatry. 2006 Sep 1;60(5):432–5. doi: 10.1016/j.biopsych.2006.02.004 16581033

[18] Sajatovic M, Forester BP, Gildengers A, Mulsant BH. Aging changes and medical complexity in late-life bipolar disorder: emerging research findings that may help advance care. Neuropsychiatry (London). 2013 Dec 12;3(6):621. doi: 10.2217/NPY.13.78 24999372 PMC4078873

[19] Winkler AM, Kochunov P, Blangero J, Almasy L, Zilles K, Fox PT, Cortical Thickness or Grey Matter Volume? The Importance of Selecting the Phenotype for Imaging Genetics Studies. Neuroimage. 2010 Nov 11;53(3):1135. doi: 10.1016/J.NEUROIMAGE.2009.12.028 20006715 PMC2891595

[20] Blumberg HP, Kaufman J, Martin A, Whiteman R, Zhang JH, Gore JC, Amygdala and Hippocampal Volumes in Adolescents and Adults with Bipolar Disorder. Arch Gen Psychiatry. 2003 Dec;60(12):1201–8. doi: 10.1001/archpsyc.60.12.1201 14662552

[21] Kalmar JH, Wang F, Chepenik LG, Womer FY, Jones MM, Pittman B, Relation between amygdala structure and function in adolescents with bipolar disorder. J Am Acad Child Adolesc Psychiatry. 2009 Jun 1;48(6):636–42. doi: 10.1097/CHI.0b013e31819f6fbc 19454919 PMC2867040

[22] Mwangi B, Spiker D, Zunta-Soares GB, Soares JC. Prediction of pediatric bipolar disorder using neuroanatomical signatures of the amygdala. Bipolar Disord. 2014 Nov 1;16(7):713–21. doi: 10.1111/bdi.12222 24917530 PMC4234406

[23] Hibar DP, Westlye LT, Van Erp TGM, Rasmussen J, Leonardo CD, Faskowitz J, Subcortical volumetric abnormalities in bipolar disorder. Mol Psychiatry. 2016 Dec;21(12):1710–6. doi: 10.1038/mp.2015.227 26857596 PMC5116479

[24] Womer FY, Wang L, Alpert KI, Smith MJ, Csernansky JG, Barch DM, Basal ganglia and thalamic morphology in schizophrenia and bipolar disorder. Psychiatry Res Neuroimaging. 2014 Aug 30;223(2):75–83. doi: 10.1016/J.PSCYCHRESNS.2014.05.017 PMC4112520 24957866

[25] Lee DK, Lee H, Park K, Joh E, Kim CE, Ryu S. Common gray and white matter abnormalities in schizophrenia and bipolar disorder. PLoS One. 2020 May 1;15(5):e0232826. doi: 10.1371/JOURNAL.PONE.0232826 32379845 PMC7205291

[26] Wang X, Luo Q, Tian F, Cheng B, Qiu L, Wang S, Brain grey-matter volume alteration in adult patients with bipolar disorder under different conditions: A voxel-based meta-analysis. J Psychiatry Neurosci. 2019 Mar 1;44(2):89–101. doi: 10.1503/jpn.180002 30354038 PMC6397036

[27] Qi Z, Wang J, Gong J, Su T, Fu S, Huang L, Common and specific patterns of functional and structural brain alterations in schizophrenia and bipolar disorder: A multimodal voxel-based meta-analysis. J Psychiatry Neurosci. 2022 Feb 23;47(1):E32–47. doi: 10.1503/JPN.210111/TAB-RELATED-CONTENT 35105667 PMC8812718

[28] Chen L, Wang Y, Niu C, Zhong S, Hu H, Chen P, Common and distinct abnormal frontal-limbic system structural and functional patterns in patients with major depression and bipolar disorder. NeuroImage Clin. 2018 Jan 1;20:42–50. doi: 10.1016/J.NICL.2018.07.002 30069426 PMC6067086

[29] Sato J, Hirano Y, Hirakawa N, Takahashi J, Oribe N, Kuga H, Lower Hippocampal Volume in Patients with Schizophrenia and Bipolar Disorder: A Quantitative MRI Study. J Pers Med. 2021 Feb 1;11(2):1–14. doi: 10.3390/JPM11020121 PMC7918861 33668432

[30] Blond BN, Fredericks CA, Blumberg HP. Functional neuroanatomy of bipolar disorder: Structure, function, and connectivity in an amygdala-anterior paralimbic neural system. Bipolar Disord. 2012 Jun 1;14(4):340–55. doi: 10.1111/j.1399-5618.2012.01015.x 22631619 PMC3880745

[31] Kalmar JH, Wang F, Spencer L, Edmiston E, Lacadie CM, Martin A, Preliminary evidence for progressive prefrontal abnormalities in adolescents and young adults with bipolar disorder. J Int Neuropsychol Soc. 2009;15(3):476–81. doi: 10.1017/S1355617709090584 19402934 PMC2852397

[32] Najt P, Wang F, Spencer L, Johnston JAY, Cox Lippard ET, Pittman BP, Anterior cortical development during adolescence in bipolar disorder. Biol Psychiatry. 2016 Feb 15;79(4):303–10. doi: 10.1016/j.biopsych.2015.03.026 26033826 PMC4595154

[33] Wise T, Radua J, Via E, Cardoner N, Abe O, Adams TM, Common and distinct patterns of grey-matter volume alteration in major depression and bipolar disorder: evidence from voxel-based meta-analysis. Mol Psychiatry. 2017 Oct;22(10):1455–63. doi: 10.1038/mp.2016.72 27217146 PMC5622121

[34] Hibar DP, Westlye LT, Doan NT, Jahanshad N, Cheung JW, Ching CRK, Cortical abnormalities in bipolar disorder: An MRI analysis of 6503 individuals from the ENIGMA Bipolar Disorder Working Group. Mol Psychiatry. 2018 Apr;23(4):932–42. doi: 10.1038/mp.2017.73 28461699 PMC5668195

[35] Selvaraj S, Arnone D, Job D, Stanfield A, Farrow TFD, Nugent AC, Grey matter differences in bipolar disorder: A meta-analysis of voxel-based morphometry studies. Bipolar Disorders. 2012 Mar;14(2):135–45. doi: 10.1111/j.1399-5618.2012.01000.x 22420589

[36] Abé C, Ekman CJ, Sellgren C, Petrovic P, Ingvar M, Landén M. Cortical thickness, volume and surface area in patients with bipolar disorder types I and II. J Psychiatry Neurosci. 2016 Jul 1;41(4):240–50. doi: 10.1503/JPN.150093 26645741 PMC4915933

[37] Hartberg CB, Sundet K, Rimol LM, Haukvik UK, Lange EH, Nesvåg R, Brain Cortical Thickness and Surface Area Correlates of Neurocognitive Performance in Patients with Schizophrenia, Bipolar Disorder, and Healthy Adults. J Int Neuropsychol Soc. 2011;17(6):1080–93. doi: 10.1017/S1355617711001081 22013998

[38] Wang F, Kalmar JH, Womer FY, Edmiston EE, Chepenik LG, Chen R, Olfactocentric paralimbic cortex morphology in adolescents with bipolar disorder. Brain. 2011 Jul;134(7):2005–12. doi: 10.1093/brain/awr124 21666263 PMC3122371

[39] Rimol LM, Nesvåg R, Hagler DJ, Bergmann Ø, Fennema-Notestine C, Hartberg CB, Cortical Volume, Surface Area, and Thickness in Schizophrenia and Bipolar Disorder. Biol Psychiatry. 2012 Mar 15;71(6):552–60. doi: 10.1016/J.BIOPSYCH.2011.11.026 22281121

[40] Madre M, Canales-Rodríguez EJ, Fuentes-Claramonte P, Alonso-Lana S, Salgado-Pineda P, Guerrero-Pedraza A, Structural abnormality in schizophrenia versus bipolar disorder: A whole brain cortical thickness, surface area, volume and gyrification analyses. NeuroImage Clin. 2020 Jan 1;25:102131. doi: 10.1016/J.NICL.2019.102131 31911343 PMC6948361

[41] Beyer JL, Kuchibhatla M, Payne ME, MacFall J, Cassidy F, Krishnan KRR. Gray and white matter brain volumes in older adults with bipolar disorder. Int J Geriatr Psychiatry. 2009 Dec;24(12):1445–52. doi: 10.1002/gps.2285 19452498 PMC4441218

[42] Delvecchio G, Mandolini GM, Arighi A, Prunas C, Mauri CM, Pietroboni AM, Structural and metabolic cerebral alterations between elderly bipolar disorder and behavioural variant f rontotemporal dementia: A combined MRI-PET study. Aust N Z J Psychiatry. 2019 May;53(5):413–23. doi: 10.1177/0004867418815976 30545239

[43] Haller S, Xekardaki A, Delaloye C, Canuto A, Lövblad KO, Gold G, Combined analysis of grey matter voxel-based morphometry and white matter tract-based spatial statistics in late-life bipolar disorder. J Psychiatry Neurosci. 2011 Nov;36(6):391–401. doi: 10.1503/jpn.100140 21284917 PMC3201993

[44] Wijeratne C, Sachdev S, Wen W, Piguet O, Lipnicki DM, Malhi GS, Hippocampal and amygdala volumes in an older bipolar disorder sample. Int Psychogeriatrics. 2013 Jan;25(1):54–60. doi: 10.1017/S1041610212001469 22929183

[45] Zung S, Souza-Duran FL, Soeiro-De-souza MG, Uchida R, Bottino CM, Busatto GF, The influence of lithium on hippocampal volume in elderly bipolar patients: a study using voxel-based morphometry. Transl Psychiatry. 2016 Jun 28;6(6):e846. doi: 10.1038/tp.2016.97 27351600 PMC4931614

[46] Rej S, Butters MA, Aizenstein HJ, Begley A, Tsay J, Reynolds CF, Neuroimaging and neurocognitive abnormalities associated with bipolar disorder in old age. Int J Geriatr Psychiatry. 2014 Apr;29(4):421–7. doi: 10.1002/gps.4021 24006234 PMC3947373

[47] Delaloye C, Moy G, De Bilbao F, Weber K, Baudois S, Haller S, Longitudinal analysis of cognitive performances and structural brain changes in late-life bipolar disorder. Int J Geriatr Psychiatry. 2011 Dec;26(12):1309–18. doi: 10.1002/gps.2683 21394788

[48] Delaloye C, De Bilbao F, Moy G, Baudois S, Weber K, Campos L, Neuroanatomical and neuropsychological features of euthymic patients with bipolar disorder. Am J Geriatr Psychiatry. 2009 Dec;17(12):1012–21. doi: 10.1097/JGP.0b013e3181b7f0e2 20104058

[49] Baez S, Pinasco C, Roca M, Ferrari J, Couto B, García-Cordero I, Brain structural correlates of executive and social cognition profiles in behavioral variant frontotemporal dementia and elderly bipolar disorder. Neuropsychologia. 2019 Mar 18;126:159–69. doi: 10.1016/j.neuropsychologia.2017.02.012 28219620

[50] Lippard ETC, Johnston JAY, Spencer L, Quatrano S, Fan S, Sankar A, Preliminary examination of gray and white matter structure and longitudinal structural changes in frontal systems associated with future suicide attempts in adolescents and young adults with mood disorders. J Affect Disord. 2019 Feb 15;245:1139–48. doi: 10.1016/J.JAD.2018.11.097 30699858 PMC6487887

[51] Serafini G, Pompili M, Innamorati M, Girardi N, Strusi L, Amore M, The impact of periventricular white matter lesions in patients with bipolar disorder type I. CNS Spectr. 2016 Jun 12;21(1):23–34. doi: 10.1017/S1092852913000825 24411553

[52] Marlinge E, Bellivier F, Houenou J, Henri Mondor-Albert Chenevier G, Houenou AP-HPJ. White matter alterations in bipolar disorder: potential for drug discovery and development. Bipolar Disord. 2014 Mar 1;16(2):97–112. doi: 10.1111/BDI.12135 24571279

[53] Tamashiro JH, Zung S, Zanetti MV, de Castro CC, Vallada H, Busatto GF, Increased rates of white matter hyperintensities in late-onset bipolar disorder. Bipolar Disord. 2008 Nov;10(7):765–75. doi: 10.1111/j.1399-5618.2008.00621.x 19032708

[54] Gunde E, Blagdon R, Hajek T. White matter hyperintensities—from medical comorbidities to bipolar disorders and back. Ann Med. 2011 Dec;43(8):571–80. doi: 10.3109/07853890.2011.595733 21749303 PMC4831903

[55] Nortje G, Stein DJ, Radua J, Mataix-Cols D, Horn N. Systematic review and voxel-based meta-analysis of diffusion tensor imaging studies in bipolar disorder. J Affect Disord. 2013 Sep 5;150(2):192–200. doi: 10.1016/J.JAD.2013.05.034 23810479

[56] Mahon K, Burdick KE, Szeszko PR. A role for white matter abnormalities in the pathophysiology of bipolar disorder. Neurosci Biobehav Rev. 2010 Mar 1;34(4):533–54. doi: 10.1016/J.NEUBIOREV.2009.10.012 19896972 PMC2847441

[57] Favre P, Pauling M, Stout J, Hozer F, Sarrazin S, Abé C, Widespread white matter microstructural abnormalities in bipolar disorder: evidence from mega- and meta-analyses across 3033 individuals. Neuropsychopharmacology. 2019 Dec;44(13):2285–93. doi: 10.1038/s41386-019-0485-6 31434102 PMC6898371

[58] Barysheva M, Jahanshad N, Foland-Ross L, Altshuler LL, Thompson PM. White matter microstructural abnormalities in bipolar disorder: A whole brain diffusion tensor imaging study. Neuroimage. 2013;2(1):558. doi: 10.1016/J.NICL.2013.03.016 24179807 PMC3777761

[59] Benedetti F, Yeh PH, Bellani M, Radaelli D, Nicoletti MA, Poletti S, Disruption of white matter integrity in bipolar depression as a possible structural marker of illness. Biol Psychiatry. 2011 Feb 15;69(4):309–17. doi: 10.1016/J.BIOPSYCH.2010.07.028 20926068

[60] Lu LH, Zhou XJ, Keedy SK, Reilly JL, Sweeney JA. White matter microstructure in untreated first episode bipolar disorder with psychosis: comparison with schizophrenia. Bipolar Disord. 2011 Nov;13(7-8):604–13. doi: 10.1111/J.1399-5618.2011.00958.X 22085473 PMC3612986

[61] Wise T, Radua J, Nortje G, Cleare AJ, Young AH, Arnone D. Voxel-Based Meta-Analytical Evidence of Structural Disconnectivity in Major Depression and Bipolar Disorder. Biol Psychiatry. 2016 Feb 15;79(4):293–302. doi: 10.1016/J.BIOPSYCH.2015.03.004 25891219

[62] Zhang R, Jiang X, Chang M, Wei S, Tang Y, Wang F. White matter abnormalities of corpus callosum in patients with bipolar disorder and suicidal ideation. Ann Gen Psychiatry. 2019 Sep 10;18:20. doi: 10.1186/S12991-019-0243-5 31528196 PMC6737682

[63] Wang F, Kalmar JH, Edmiston E, Chepenik LG, Bhagwagar Z, Spencer L, Abnormal Corpus Callosum Integrity in Bipolar Disorder: A Diffusion Tensor Imaging Study. Biol Psychiatry. 2008 Oct 15;64(8):730–3. doi: 10.1016/J.BIOPSYCH.2008.06.001 18620337 PMC2586998

[64] Prunas C, Delvecchio G, Perlini C, Barillari M, Ruggeri M, Altamura AC, Diffusion imaging study of the Corpus Callosum in bipolar disorder. Psychiatry Res Neuroimaging. 2018 Jan 30;271:75–81. doi: 10.1016/J.PSCYCHRESNS.2017.11.001 29129544

[65] Caseras X, Murphy K, Lawrence NS, Fuentes-Claramonte P, Watts J, Jones DK, Emotion regulation deficits in euthymic bipolar I versus bipolar II disorder: a functional and diffusion-tensor imaging study. Bipolar Disord. 2015 Aug 1;17(5):461. doi: 10.1111/BDI.12292 25771686 PMC4672703

[66] Foley SF, Bracher-Smith M, Tansey KE, Harrison JR, Parker GD, Caseras X. Fractional anisotropy of the uncinate fasciculus and cingulum in bipolar disorder type I, type II, unaffected siblings and healthy controls. Br J Psychiatry. 2018 Sep 1;213(3):548–54. doi: 10.1192/BJP.2018.101 30113288 PMC6130806

[67] Li X, Lu W, Zhang R, Zou W, Gao Y, Chen K, Integrity of the uncinate fasciculus is associated with the onset of bipolar disorder: a 6-year followed-up study. Transl Psychiatry. 2021 Feb 5;11(1):1–7. doi: 10.1038/s41398-021-01222-z 33547277 PMC7864939

[68] Lin F, Weng S, Xie B, Wu G, Lei H. Abnormal frontal cortex white matter connections in bipolar disorder: a DTI tractography study. J Affect Disord. 2011 Jun;131(1-3):299–306. doi: 10.1016/J.JAD.2010.12.018 21236494

[69] Xu E, Nguyen L, Hu R, Stavish CM, Leibenluft E, Linke JO. The uncinate fasciculus in individuals with and at risk for bipolar disorder: A meta-analysis. J Affect Disord. 2022 Jan 15;297:208–16. doi: 10.1016/J.JAD.2021.10.045 34699854 PMC8631233

[70] Coad BM, Postans M, Hodgetts CJ, Muhlert N, Graham KS, Lawrence AD. Structural connections support emotional connections: Uncinate Fasciculus microstructure is related to the ability to decode facial emotion expressions. Neuropsychologia. 2020 Aug;145:106562. doi: 10.1016/J.NEUROPSYCHOLOGIA.2017.11.006 29122609 PMC7534036

[71] Almeida OP, Dols A, Blanken MAJT, Rej S, Blumberg HP, Villa L, Physical Health Burden Among Older Men and Women With Bipolar Disorder: Results From the Gage-Bd Collaboration. Am J Geriatr Psychiatry. 2022 Jun 1;30(6):727–32. doi: 10.1016/J.JAGP.2021.12.006 34980553

[72] Lala SV, Sajatovic M Medical and psychiatric comorbidities among elderly individuals with bipolar disorder: A literature review. J Geriatr Psychiatry Neurol. 2012 Mar 30;25(1):20–5. doi: 10.1177/0891988712436683 22467842

[73] Goldstein BI, Baune BT, Bond DJ, Chen PH, Eyler L, Fagiolini A, Call to action regarding the vascular-bipolar link: A report from the Vascular Task Force of the International Society for Bipolar Disorders. Bipolar Disord. 2020 Aug 1;22(5):440–60. doi: 10.1111/BDI.12921 32356562 PMC7522687

[74] Versace A, Almeida JRC, Hassel S, Walsh ND, Novelli M, Klein CR, Elevated left and reduced right orbitomedial prefrontal fractional anisotropy in adults with bipolar disorder revealed by tract-based spatial statistics. Arch Gen Psychiatry. 2008 Sep;65(9):1041–52. doi: 10.1001/ARCHPSYC.65.9.1041 18762590 PMC2730162

[75] Xekardaki A, Giannakopoulos P, Haller S. White Matter Changes in Bipolar Disorder, Alzheimer Disease, and Mild Cognitive Impairment: New Insights from DTI. J Aging Res. 2011;2011:286564. doi: 10.4061/2011/286564 22187647 PMC3236486

[76] Poletti S, Bollettini I, Mazza E, Locatelli C, Radaelli D, Vai B, Cognitive performances associate with measures of white matter integrity in bipolar disorder. J Affect Disord. 2015 Mar 15;174:342–52. doi: 10.1016/J.JAD.2014.12.030 25553397

[77] Magioncalda P, Martino M, Conio B, Piaggio N, Teodorescu R, Escelsior A, Patterns of microstructural white matter abnormalities and their impact on cognitive dysfunction in the various phases of type I bipolar disorder. J Affect Disord. 2016 Mar 15;193:39–50. doi: 10.1016/J.JAD.2015.12.050 26766032

[78] Masuda Y, Okada G, Takamura M, Shibasaki C, Yoshino A, Yokoyama S, White matter abnormalities and cognitive function in euthymic patients with bipolar disorder and major depressive disorder. Brain Behav. 2020 Dec 1;10(12):e01868. doi: 10.1002/BRB3.1868 33009714 PMC7749556

[79] Solé B, Jiménez E, Torrent C, Reinares M, Del Mar Bonnin C, Torres I, Cognitive Impairment in Bipolar Disorder: Treatment and Prevention Strategies. Int J Neuropsychopharmacol. 2017 Aug 1;20(8):670–80. doi: 10.1093/IJNP/PYX032 28498954 PMC5570032

[80] Manji HK, Moore GJ, Chen G. Clinical and preclinical evidence for the neurotrophic effects of mood stabilizers: implications for the pathophysiology and treatment of manic-depressive illness. Biol Psychiatry. 2000 Oct 15;48(8):740–54. doi: 10.1016/S0006-3223(00)00979-3 11063971

[81] Foland LC, Altshuler LL, Sugar CA, Lee AD, Leow AD, Townsend J, Increased volume of the amygdala and hippocampus in bipolar patients treated with lithium. Neuroreport. 2008 Jan 1;19(2):221. doi: 10.1097/WNR.0B013E3282F48108 18185112 PMC3299336

[82] Hozer F, Sarrazin S, Laidi C, Favre P, Pauling M, Cannon D, Lithium prevents grey matter atrophy in patients with bipolar disorder: an international multicenter study. Psychol Med. 2021 May 1;51(7):1201–10. doi: 10.1017/S0033291719004112 31983348

[83] Abramovic L, Boks MPM, Vreeker A, Verkooijen S, van Bergen AH, Ophoff RA, White matter disruptions in patients with bipolar disorder. Eur Neuropsychopharmacol. 2018 Jun 1;28(6):743. doi: 10.1016/J.EURONEURO.2018.01.001 29779901 PMC6008233

[84] Haarman BCM, Riemersma-Van Der Lek RF, Burger H, De Groot JC, Drexhage HA, Nolen WA, Diffusion tensor imaging in euthymic bipolar disorder - A tract-based spatial statistics study. J Affect Disord. 2016 Oct 1;203:281–91. doi: 10.1016/J.JAD.2016.05.040 27317921

[85] Espanhol JCL, Vieira-Coelho MA. Effects of lithium use on the white matter of patients with bipolar disorder—a systematic review. Nord J Psychiatry. 2022 Jan;76(1):1–11. doi: 10.1080/08039488.2021.1921264 33969798

[86] Gildengers AG, Butters MA, Aizenstein HJ, Marron MM, Emanuel J, Anderson SJ, Longer lithium exposure is associated with better white matter integrity in older adults with bipolar disorder. Bipolar Disord. 2015 May;17(3):248–56. doi: 10.1111/bdi.12260 25257942 PMC4374042

[87] Gerhard T, Devanand DP, Huang C, Crystal S, Olfson M. Lithium treatment and risk for dementia in adults with bipolar disorder: Population-based cohort study. Br J Psychiatry. 2015 Jul 1;207(1):46–51. doi: 10.1192/BJP.BP.114.154047 25614530

[88] Chen P, Eyler LT, Gildengers A, Beunders AJ, Blumberg HP, Briggs FB, Demographic and Clinical Characteristics of Antipsychotic Drug-Treated Older Adults with Bipolar Disorder from the Global Aging & Geriatric Experiments in Bipolar Disorder Database (GAGE-BD). Psychopharmacol Bull. 2022 May 1;52(2):8–33. 10.64719/pb.4431 PMC9172555 35721813

[89] Birner A, Bengesser SA, Seiler S, Dalkner N, Queissner R, Platzer M, Total gray matter volume is reduced in individuals with bipolar disorder currently treated with atypical antipsychotics. J Affect Disord. 2020 Jan 1;260:722–7. doi: 10.1016/J.JAD.2019.09.068 31563071

[90] Kyriakopoulos M, Samartzis L, Dima D, Hayes D, Corrigall R, Barker G, Does antipsychotic medication affect white matter in schizophrenia and bipolar disorder? a review of diffusion tensor imaging literature. Eur Psychiatry. 2011 Mar;26(S2):1280. doi: 10.1016/S0924-9338(11)72985-6

[91] Gildengers AG, Chung KH, Huang SH, Begley A, Aizenstein HJ, Tsai SY. Neuroprogressive effects of lifetime illness duration in older adults with bipolar disorder. Bipolar Disord. 2014 Sep 1;16(6):617–23. doi: 10.1111/BDI.12204 24716786 PMC4149863

[92] Huang YC, Wang LJ, Tseng PT, Hung CF, Lin PY. Leukocyte telomere length in patients with bipolar disorder: An updated meta-analysis and subgroup analysis by mood status. Psychiatry Res. 2018 Dec;270:41–9. doi: 10.1016/j.psychres.2018.09.035 30243131

[93] Brown NC, Andreazza AC, Young LT. An updated meta-analysis of oxidative stress markers in bipolar disorder. Psychiatry Res. 2014 Aug 15;218(1-2):61–8. doi: 10.1016/j.psychres.2014.04.005 24794031

[94] Chang CC, Jou SH, Lin TT, Liu CS. Mitochondrial DNA variation and increased oxidative damage in euthymic patients with bipolar disorder. Psychiatry Clin Neurosci. 2014 Jul;68(7):551–7. doi: 10.1111/pcn.12163 24447331

[95] Lemaitre H, Goldman AL, Sambataro F, Verchinski BA, Meyer-Lindenberg A, Weinberger DR, Normal age-related brain morphometric changes: Nonuniformity across cortical thickness, surface area and gray matter volume? Neurobiol Aging. 2012 Mar;33(3):617.e1–9. doi: 10.1016/j.neurobiolaging.2010.07.013 PMC3026893 20739099

[96] Hogstrom LJ, Westlye LT, Walhovd KB, Fjeli AM. The structure of the cerebral cortex across adult life: Age-related patterns of surface area, thickness, and gyrification. Cereb Cortex. 2013 Nov;23(11):2521–30. doi: 10.1093/cercor/bhs231 22892423

[97] Hutton C, Draganski B, Ashburner J, Weiskopf N. A comparison between voxel-based cortical thickness and voxel-based morphometry in normal aging. Neuroimage. 2009 Nov 1;48(2):371–80. doi: 10.1016/j.neuroimage.2009.06.043 19559801 PMC2741580

[98] Rathee R, Rallabandi VPS, Roy PK. Age-Related Differences in White Matter Integrity in Healthy Human Brain: Evidence from Structural MRI and Diffusion Tensor Imaging. Magn Reson Insights. 2016 Jan;9:9. doi: 10.4137/MRI.S39666 27279747 PMC4898444

[99] Billiet T, Vandenbulcke M, Mädler B, Peeters R, Dholiander T, Zhang H, Age-related microstructural differences quantified using myelin water imaging and advanced diffusion MRI. Neurobiol Aging. 2015 Jun 1;36(6):2107–21. doi: 10.1016/J.NEUROBIOLAGING.2015.02.029 25840837

[100] Nenadić I, Dietzek M, Langbein K, Sauer H, Gaser C. BrainAGE score indicates accelerated brain aging in schizophrenia, but not bipolar disorder. Psychiatry Res Neuroimaging. 2017 Aug 30;266:86–9. doi: 10.1016/j.pscychresns.2017.05.006 28628780

[101] Shahab S, Mulsant BH, Levesque ML, Calarco N, Nazeri A, Wheeler AL, Brain structure, cognition, and brain age in schizophrenia, bipolar disorder, and healthy controls. Neuropsychopharmacology. 2019 Apr;44(5):898–906. doi: 10.1038/s41386-018-0298-z 30635616 PMC6461913

[102] Sani G, Chiapponi C, Piras F, Ambrosi E, Simonetti A, Danese E, Gray and white matter trajectories in patients with bipolar disorder. Bipolar Disord. 2016 Feb 1;18(1):52–62. doi: 10.1111/BDI.12359 26782273

[103] Tønnesen S, Kaufmann T, Doan NT, Alnæs D, Córdova-Palomera A, Meer D van der, White matter aberrations and age-related trajectories in patients with schizophrenia and bipolar disorder revealed by diffusion tensor imaging. Sci Rep. 2018 Sep 20;8(1):1–14. doi: 10.1038/s41598-018-32355-9 30237410 PMC6147807

[104] Tønnesen S, Kaufmann T, de Lange AMG, Richard G, Doan NT, Alnæs D, Brain Age Prediction Reveals Aberrant Brain White Matter in Schizophrenia and Bipolar Disorder: A Multisample Diffusion Tensor Imaging Study. Biol Psychiatry Cogn Neurosci Neuroimaging. 2020 Dec 1;5(12):1095–103. doi: 10.1016/J.BPSC.2020.06.014 32859549

[105] Ballester PL, Romano MT, de Azevedo Cardoso T, Hassel S, Strother SC, Kennedy SH, Brain age in mood and psychotic disorders: a systematic review and meta-analysis. Acta Psychiatr Scand. 2022 Jan 1;145(1):42–55. doi: 10.1111/ACPS.13371 34510423

[106] Vasudev A, Thomas A. “Bipolar disorder” in the elderly: what’s in a name? Maturitas. 2010 Jul;66(3):231–5. doi: 10.1016/J.MATURITAS.2010.02.013 20307944

[107] Zanetti MV, Cordeiro Q, Busatto GF. Late onset bipolar disorder associated with white matter hyperintensities: A pathophysiological hypothesis. Prog Neuro-Psychopharmacology Biol Psychiatry. 2007 Mar 30;31(2):551–6. doi: 10.1016/J.PNPBP.2006.10.004 17107742

[108] Alves GS, Knöchel C, Paulitsch MA, Reinke B, Carvalho AF, Feddern R, White matter microstructural changes and episodic memory disturbances in late-onset bipolar disorder. Front Psychiatry. 2018 Oct 9;9:480. doi: 10.3389/fpsyt.2018.00480 30356890 PMC6190894

[109] Bellivier F, Golmard JL, Rietschel M, Schulze TG, Malafosse A, Preisig M, Age at onset in bipolar I affective disorder: Further evidence for three subgroups. Am J Psychiatry. 2003 May;160(5):999–1001. doi: 10.1176/appi.ajp.160.5.999 12727708

[110] Tozzi F, Manchia M, Galwey NW, Severino G, Del Zompo M, Day R, Admixture analysis of age at onset in bipolar disorder. Psychiatry Res. 2011 Jan 30;185(1-2):27–32. doi: 10.1016/j.psychres.2009.11.025 20580841

[111] Sarrazin S, Cachia A, Hozer F, McDonald C, Emsell L, Cannon DM, Neurodevelopmental subtypes of bipolar disorder are related to cortical folding patterns: An international multicenter study. Bipolar Disord. 2018; doi: 10.1111/bdi.12664 PMC6516086 29981196

[112] Penttilä J, Cachia A, Martinot JL, Ringuenet D, Wessa M, Houenou J, Cortical folding difference between patients with early-onset and patients with intermediate-onset bipolar disorder. Bipolar Disord. 2009 Jun;11(4):361–70. doi: 10.1111/j.1399-5618.2009.00683.x 19500089

[113] Schouws SNTM, Comijs HC, Stek ML, Dekker J, Oostervink F, Naarding P, Cognitive impairment in early and late bipolar disorder. Am J Geriatr Psychiatry. 2009 Jun;17(6):508–15. doi: 10.1097/JGP.0b013e31819e2d50 19461259

[114] Martino DJ, Strejilevich SA, Manes F. Neurocognitive functioning in early-onset and late-onset older patients with euthymic bipolar disorder. Int J Geriatr Psychiatry. 2013 Feb;28(2):142–8. doi: 10.1002/gps.3801 22451354

[115] Sarmento SMDS, Bittencourt L, De Mendoncą Filho EJ, Abreu N, Tavares De Lacerda AL, Miranda-Scippa Â. Neurocognitive Impairment in Bipolar Disorder and Associated Factors: Using Population-based Norms and a Strict Criterion for Impairment Definition. Cogn Behav Neurol. 2020 Jun;33(2):103–12. doi: 10.1097/WNN.0000000000000231 32496295

[116] Liu T, Zhong S, Wang B, Liao X, Lai S, Jia Y. Similar profiles of cognitive domain deficits between medication-naïve patients with bipolar II depression and those with major depressive disorder. J Affect Disord. 2019 Jan 15;243:55–61. doi: 10.1016/j.jad.2018.05.040 30227315

[117] Martino DJ, Marengo E, Igoa A, Strejilevich SA. Neurocognitive heterogeneity in older adults with bipolar disorders. Psychiatry Res. 2018 Apr;262:510–2. doi: 10.1016/j.psychres.2017.09.035 28942955

[118] Besga A, Martinez-Cengotitabengoa M, González-Ortega I, Gutierrez M, Barbeito S, Gonzalez-Pinto A. The role of white matter damage in late onset bipolar disorder. Maturitas. 2011 Oct 1;70(2):160–3. doi: 10.1016/J.MATURITAS.2011.07.005 21872409

[119] Liu T, Lipnicki DM, Zhu W, Tao D, Zhang C, Cui Y, Cortical gyrification and sulcal spans in early stage Alzheimer’s disease. PLoS One. 2012;7(2):e31083. doi: 10.1371/journal.pone.0031083 22363554 PMC3283590

[120] Cai K, Xu H, Guan H, Zhu W, Jiang J, Cui Y, Identification of early-stage Alzheimer’s disease using sulcal morphology and other common neuroimaging indices. PLoS One. 2017 Jan 27;12(1):e0170875. doi: 10.1371/journal.pone.0170875 28129351 PMC5271367

[121] Sajatovic M, Eyler LT, Rej S, Almeida OP, Blumberg HP, Forester BP, The Global Aging & Geriatric Experiments in Bipolar Disorder Database (GAGE-BD) project: Understanding older-age bipolar disorder by combining multiple datasets. Bipolar Disord. 2019 Nov 1;21(7):642–9. doi: 10.1111/BDI.12795 31081573

